# Nonpharmacological Interventions to Reduce Behavioral and Psychological Symptoms of Dementia: A Systematic Review

**DOI:** 10.1155/2015/218980

**Published:** 2015-11-29

**Authors:** Alexandra Martini de Oliveira, Marcia Radanovic, Patrícia Cotting Homem de Mello, Patrícia Cardoso Buchain, Adriana Dias Barbosa Vizzotto, Diego L. Celestino, Florindo Stella, Catherine V. Piersol, Orestes V. Forlenza

**Affiliations:** ^1^Laboratory of Neuroscience (LIM-27), Department and Institute of Psychiatry, Faculty of Medicine, University of São Paulo, 05403-010 São Paulo, SP, Brazil; ^2^Occupational Therapy Service, Institute of Psychiatry, Faculty of Medicine, University of São Paulo, 05403-010 São Paulo, SP, Brazil; ^3^Biosciences Institute, Universidade Estadual Paulista (UNESP), 13506-900 Rio Claro, SP, Brazil; ^4^Department of Occupational Therapy, Clinical Director, Jefferson Elder Care, Thomas Jefferson University, Philadelphia, PA 19107, USA

## Abstract

*Introduction*. Behavioral and psychological symptoms of dementia (BPSD) are defined as a group of symptoms of disturbed perceptive thought content, mood, or behavior that include agitation, depression, apathy, repetitive questioning, psychosis, aggression, sleep problems, and wandering. Care of patients with BPSD involves pharmacological and nonpharmacological interventions. We reviewed studies of nonpharmacological interventions published in the last 10 years.* Methods*. We performed a systematic review in Medline and Embase databases, in the last 10 years, until June 2015. Key words used were (1) non-pharmacological interventions, (2) behavioral symptoms, (3) psychological symptoms, and (4) dementia.* Results*. We included 20 studies published in this period. Among these studies, program activities were more frequent (five studies) and the symptoms more responsive to the interventions were agitation.* Discussion*. Studies are heterogeneous in many aspects, including size sample, intervention, and instruments of measures.* Conclusion*. Nonpharmacological interventions are able to provide positive results in reducing symptoms of BPSD. Most studies have shown that these interventions have important and significant efficacy.

## 1. Introduction

The term BPSD stands for “behavioral and psychological symptoms of dementia” and has been used to describe a group of heterogeneous symptoms that arise in the course of dementia and are distressing and difficult to manage, both for caregivers and health professionals [1]. One or more symptoms of BPSD will affect up to 90% of patients with dementia during the disease course [2–4]. BPSD include many different behaviors such as screaming (disruptive vocalization), restlessness, repetitive questions, wandering, and apathy. As dementia is a progressive disease, BPSD worsen over time, requiring higher support and increasing cost of care [5]. BPSD have been associated with a poorer prognosis, a more rapid rate of cognitive decline, and illness progression [6], greater impairment in activities of daily living (ADLs) [6], and increased institutionalization at hospitals or residential care facilities [2, 7]. The frequency and severity of these symptoms have been strongly correlated with caregiver burden, reducing quality of life of patients and their caregivers [8].

Psychotropic medications have modest efficacy and can lead to undesired side effects [9–11] but they are frequently used to treat BPSD [12–15]. An increasing number of medical organizations and specialist groups such as the American Geriatric Society and the American Association for Geriatric Psychiatry currently consider nonpharmacological interventions to be first line Clinical Practice, except for emergency situations, referring mostly to situations in which the patient's behavior is harmful to him/her or other persons [16–18]. According to Cohen-Mansfield [19] most professionals have some training in medication prescription for BPSD, but few are instructed about nonpharmacological interventions or receive information about their effectiveness. As a consequence, antipsychotics drugs are frequently prescribed before alternative nonpharmacological approaches are attempted, and patients are maintained in medication for long periods, which leads to increasing morbidity and mortality. This scenario may be improved if professionals involved in dementia care are better apprised of the indications and limitations for the several existing nonpharmacological therapies for BPSD.

Some studies have shown that nonpharmacological treatments pose fewer side effects, which render them as safer options [20]. Nonpharmacological alternatives, including music therapy, aromatherapy, art therapy, behavioral therapy, reality orientation, tailored activities, and physical exercises, have shown promising results for the management of BPSD [21–23]. The aim of the present review was to identify and summarize the main nonpharmacological interventions for BPSD in the treatment of patients with dementia published in the last ten years.

## 2. Methods

### 2.1. Literature Review

We systematically searched the Medline and Embase databases using the following keywords: (1) non-pharmacological interventions, (2) behavioral symptoms, (3) psychological symptoms, and (4) dementia. These terms were selected even in the absence of specific Mesh terms as to increase sensitivity. The date limits ranged from the first paper published in 2005 to June 2015. We also looked for reviews to identify relevant articles about the issue.

### 2.2. Eligibility Criteria

To be included in the review, papers had to be written in English, Spanish, or Portuguese and have appropriate description of the study design (e.g., clinical trials, interventional studies, or clinical studies). Systematic reviews, meta-analyses, case reports, and editorial letters were not included in our review.

After selection and analysis of papers according to the above-mentioned inclusion criteria, the following variables were extracted and organized: (a) Overview: study design, authors, and year of publication; (b) Demographic: total sample (number of participants) and location; and (c) Assessment of BPSD. A critical analysis was performed in order to investigate the response of patients presenting diverse symptoms of BPSD to different nonpharmacological approaches.

## 3. Results

Our initial search returned 33 references. Of these, 20 studies met the inclusion criteria and were included in our review: five on activities, four on music therapy, three on aromatherapy, three on exercises, two on light therapy, one on touch therapy, one on combination of activities, and one on cognitive rehabilitation (Table 1). A brief comment and critical overview on selected studies is presented as follows.

### 3.1. Occupational Activities

The use of activities as nonpharmacological intervention for people with dementia has shown potential benefits in quality of life and in reducing agitation and depression [37–39]. Five studies investigated the effect of activities in BPSD in patients with dementia, with three addressing the “Tailored Activities Program” (TAP). TAP is an occupational therapy intervention program that focuses on reducing undesirable behaviors associated with dementia [28]. The principle of TAP is the selection of activities that are specifically tailored to the patient according to his/her abilities, interests, and roles. The program also offers training for caregivers in order to simplify activities and to adapt them for future functional declines of the patient as well as to generalize strategies to other contexts, thus helping caregivers to develop an increased sense of self-efficacy. In the US-TAP study, Gitlin et al. [28] demonstrated reduction in the overall incidence of BPSD and specific behaviors such as shadowing, agitation, argumentation, and repetitive questioning in a sample with 60 dyads. The US-TAP interventions proved to be effective in reducing shadowing (*p* = 0.003) and behavioral occurrences (*p* = 0.009). The Australian TAP was published as “protocol-only paper” and results are not yet available [11].

### 3.2. Music Therapy

Music therapy is one of the nonpharmacological methods used to reduce BPSD [26, 40]. We found four studies about the effectiveness of music therapy for the management of BPSD. One investigated the effects of two interventions, simulated family presence and preferred music, where participants were exposed to 15-minute audiotape sessions. One group of participants heard audiotapes with a conversation about positive experiences from the past and the other was exposed to a selection of songs that the individuals used to enjoy in their youth. Both interventions proved to be effective in reducing agitation [26].

Holmes et al. [24] compared two methods of presenting music, live or prerecorded, in the treatment of apathy. Music sessions comprised three different activities of 30 minutes each. One 30-minute session consisted of silence alone, another 30-minute session consisted of background prerecorded songs, and the last one consisted of watching live music sessions. Music played during the live interactive and prerecorded sessions was the same and consisted of a mixture of favorite songs according to the age of the group. Live interactive music proved to be more effective than prerecorded music in reducing apathy in moderate and severe dementia in the short term (*p* < 0.0001). Prerecorded music did not show any efficacy in improving apathy.

Sung et al. [33] investigated the effects of group music intervention on anxiety and agitation in institutionalized elders with dementia who actively participated in a music group session of 30 minutes, twice a week, for six weeks. The 30 minutes of intervention consisted of a five-minute warm-up session with movements and breathing and a 20-minute session of active participation using percussion instruments and the last five minutes were a cool-down session with soft music. Previously the participants, caregivers, and family members were asked about patients' musical preferences and a selection of familiar songs was used in each session. Participants in the control group received routine care: activities of daily living, basic nursing care, meal provision, and some social activities (TV watching, family visiting, etc.). Results indicated that music intervention had a significant effect in reducing anxiety (*p* = 0.004).

Svansdottir and Snaedal [25] investigated the effect of music therapy in a case-control study in a sample of 38 patients and reported significant improvement in aggressiveness and anxiety. In this intervention patients and therapist sang songs chosen by the group: each song was sung twice, accompanied by a guitar and other instruments of their choice. The therapy group received 18 sessions of music therapy, each lasting 30 minutes, three times a week for 6 weeks, while the control group did not change their daily care routine. The authors concluded that music therapy significantly reduced agitation and anxiety (*p* = 0.02) in moderate and severe dementia.

### 3.3. Aromatherapy

Three studies examined the use of aromatherapy to treat BPSD. Aromatherapy involves the diffusion of aromatic oil into the environment. Two oils were used to treat agitation: lavender and melissa oil (lemon balm). Lin et al. [27] conducted a crossover randomized study, comparing lavender inhalation (A), considered as the experimental group, and sunflower inhalation (B), considered as the placebo group. Lavender is a holistic relaxant that is regarded as having carminative, antiflatulent, and anticolic properties [27]. Sunflower preparation was selected as the placebo agent as it is odorless and does not possess any known therapeutic effect. Diffusers were placed on each side of the pillow of the participant during sleep at night for at least one hour. Each participant received both treatments (A and B) for three weeks with a washout period (two weeks) between each of the treatments. In this study, lavender was effective as an adjunctive therapy in alleviating agitation in patients with dementia (*p* < 0.001).

Burns et al. [31] assessed the efficacy of melissa aromatherapy in the treatment of agitation in dementia. This study was a randomized controlled trial, and the authors reported that there were no significant differences between aromatherapy, medication (donepezil), and placebo, with the three participant groups showing improvement in the NPI and in the Pittsburgh Agitation Scale (PAS).

Yang et al. [21] investigated aromatherapy and aroma acupressure (where acupuncture points were used in the aroma acupressure protocols to treat agitation). The procedures for the acupressure consisted of the following: each acupoint was pressed for two minutes with lavender oil and warm-up exercises were performed for five minutes, no longer than 15 minutes, once per day, five days a week, for four weeks in total. In the aromatherapy group, the lavender oil was applied at five acupoints with the same procedure. The aromatherapy and control groups did not receive any other interventions. Results showed that aroma-acupressure and aromatherapy had significant effect in reducing agitation (*p* < 0.01 and *p* = 0.01, resp.) when compared to the control group.

### 3.4. Physical Exercise

There were three articles on the effect of physical exercises on BPSD. However, one of them is just a “study protocol” without results [22]. From the remaining two studies, one addresses aquatic exercises, [35] consisting of a 45-minute group session (five to seven patients who performed exercises to strengthen agility, flexibility, and balance), followed by a relaxation session. Sessions were delivered twice a week over a 12-week period with a trained instructor and some assistants. Each participant performed the exercises accompanied by an assistant. This study identified a significant decrease in the number of BPSD (*p* = 0.001), improvement in psychological wellbeing, and reduction in staff distress associated with BPSD (*p* = 0.001). The second study focused on the effect of physical exercises on BPSD, considering individually tailored walking regimen designed to become progressively intensive and to last between 20 and 30 minutes at least five times a week. Results showed that exercises did not improve BPSD but were effective in attenuating caregiver burden [15].

### 3.5. Bright Light Therapy

Bright light therapy (BLT) has been used with different results in patients with dementia, showing benefits in the management of agitation [35]. The most often reported positive results are improved night-time sleep, reduction in agitation, and improvement in cognitive performance. The study by Burns et al. [29] assessed the effect of BLT on BPSD and found that sleep quality was particularly improved in 48 patients with dementia. Their experimental group consisted of patients exposed to light with an intensity of 10,000 lux and the control group consisted of patients exposed to standard fluorescent tube light at 100 lux during two weeks for two hours per day. Agitation improved but there was no statistically significant effect.

Dowling et al. [36] tested the effects of BLT in a randomized trial, with the BLT being administered for one hour daily (Monday to Friday) for 11 weeks. One group was exposed to light in the morning period; the second group was exposed to light in the afternoon, and the third group was exposed to indoor light. There were statistically significant differences between morning light exposure and afternoon light exposure in agitation/aggression scores (*p* = 0.032) and between morning light and indoor light in aberrant motor behavior (AMB) scores at the end of intervention (*p* = 0.021).

### 3.6. Touch Therapy

Touch therapies can include massage, craniosacral techniques, or therapeutic touch. Woods and Dimond [5] investigated the effect of touch therapy on BPSD in a double-blind three-group experimental study (one group with therapeutic touch, one group with placebo therapeutic touch, and the third group without any touch intervention). The interventions consisted of two daily sessions of 5–7 minutes for three days. The experimental group (therapeutic touch) experienced a statistically significant effect in reducing behavioral symptoms when compared to the group without any touch intervention (*p* = 0.036).

### 3.7. Combined Activities

One study investigated the efficacy of a combination of nonpharmacological interventions on BPSD among older Chinese men in Taiwan [23] in a prospective study with residents in dementia care units. The combination included music therapy, orientation training, physical exercise, and art cognitive activities. All the interventions were delivered by trained occupational therapists and the frequency of interventions for the study group was twice a week for 12 weeks. Music therapy consisted of activities encouraging participants to sing, to move their arms to the rhythm of the songs, and to use simple percussion instruments. Exercise included ball games and other recreational activities designed to increase the inhabitants' activity level. Art cognitive activities included various painting activities; for example, participants were asked to color a drawing of a beach scene and at the same time a conversation about the characteristics of summer was introduced to increase orientation. The intervention group had more significant reduction than the reference group in the NPI score (*p* = 0.046), including delusion (*p* = 0.018), hallucination (*p* = 0.004), and agitation (*p* = 0.038) [23].

### 3.8. Cognitive Rehabilitation

Only one study addressed cognitive rehabilitation for BPSD [34, 41]. The impact of cognitive interventions on the BPSD is still not well known because most of studies have focused on improving global or specific cognitive functions [41]. Considering the importance of the relationship between cognitive interventions and BPSD, Brunelle-Hamann et al. [34] evaluated a cognitive program in patients with mild and moderate AD. This cognitive program consisted in a four-week home-based intervention of 45–60-minute sessions twice a week for four weeks and involved memory techniques to relearn an instrumental activity of the daily living chosen by patients and caregivers (e.g., origami, computer, and TV remote control). The level of assistance was provided according to the necessity of each participant. After interventions, there was a significant reduction of delusional symptoms with a large effect size; however, aberrant motor behavior increased significantly in the treatment condition when compared to the control group [34].

## 4. Discussion

This paper provides an overview of current evidence on the efficacy of nonpharmacological interventions to reduce BPSD published in the last 10 years. All the interventions discussed in this review were dedicated to patients and caregivers. Studies are heterogeneous regarding intervention protocols, instruments of clinical assessment, and evaluation of outcome.

Five studies investigated the effectiveness of activity programs and demonstrated positive results. However, the intervention methods varied across studies. These studies used different theoretical backgrounds and investigated the effect of personalized activities. The largest effect size was found when the treatments were tailored to participants' interests and skills [30].

Four studies assessed the impact of music therapy, using different interventions, showing positive effects. The use of familiar songs reduced anxiety [24, 33]. According to Gerdner [42, 43], music can change the focus of attention and provide an interpretable stimulus that elicits positive memories from an earlier period in the person's life, which would prevent, or alleviate, anxiety or agitation. Live interactive music presents efficacy in the short term management of apathy in patients with moderate and severe dementia, whereas prerecorded music produces a more limited effect [24].

Researchers have investigated therapeutic touch with experimental and longitudinal study designs. Woods and Dimond [5] found that therapeutic touch can be used to decrease behavioral symptoms of dementia, specifically restlessness and vocalization. The mechanism of action of therapeutic touch is still unknown.

Bright light therapy (BLT) has been increasingly studied and regarded as appropriate method to improve fluctuations in diurnal rhythms that may account for night-time disturbances and the “sundown syndrome” (confusion or agitation in the late afternoon or early evening). The BLT studies included in this review revealed significant positive effects of this intervention in BPSD, especially in agitated behavior and sleep disturbance. The most likely explanation for these effects is the influence of BLT on the melatonin system, which is implicated in the regulation of abnormal motor behavior during sleep [31].

Aromatherapy may be beneficial to agitated patients with dementia [21, 27, 31]. However, varying degrees of anosmia have been reported in people with dementia [27], which might lead to analytical bias.

Most studies included in our review focused on and reported behavioral abnormalities such as agitation. However, one study showed that an organized nonpharmacological intervention program was effective in managing both outward and intrinsic symptoms, including hallucination and delusion [23].

Studies focusing on the implementation of physical exercises programs have demonstrated reduction in BPSD and improvement of psychological wellbeing in patients with dementia. However, most studies were based on small samples, and further studies are warranted.

In our review, ten of the twenty studies indicated that nonpharmacological interventions are effective in reducing agitation. Agitation is a very common, persistent, and distressing symptom among people with moderate and severe dementia, affecting 30% of those living at home [44]. According to Livingston et al. [45], agitation in dementia is associated with poorer quality of life and impairs the engagement in daily activities and relationships. In addition to causing distress in family members and caregivers, it may precipitate institutionalization at nursing homes.

Currently, in clinical practice, pharmacological treatment of agitation is usually performed using antipsychotic drugs. However, clinical outcomes are poor and undesired side effects (including cognitive worsening, confusion, and extrapyramidal signs) are frequent, even with the use of the newer atypical drugs [14]. Therefore, nonpharmacological interventions seem to provide safer and effective alternatives for treating agitation in patients with dementia.

Regarding clinical settings, the majority of the studies (*n* = 15) included in this review focused on interventions on patients with dementia residing at long term care facilities, and their application to home-based support remains uncertain [46, 47]. According to Trivedi et al. [1], two-thirds of patients with dementia live at home and yet there is limited evidence on which methods are the most effective in this setting.

Interestingly, one study reported significant worsening on BPSD. Brunelle-Hamann et al. [34], in a single blind, block-randomized and crossover-controlled study, investigated the impact of cognitive rehabilitation program on BPSD in AD patients. The results revealed that aberrant motor behaviors increased significantly in the treatment condition when compared to the control condition. The proposed hypothesis was that, during the rehabilitation intervention, as the dementia progresses, AD patients gradually lose their coping abilities and perceive their environment as more stressful.

Some limitations of the included studies need to be addressed. In terms of BPSD measures, most scales rely on information provided by caregivers, being thus subjected to the interference of variables such as caregiver's burden, personality, and even his/her ability to perceive changes in patients' behavior. However, the studies addressed in our review employed instruments that are validated and widely used in dementia research. Our review encompassed single-blind, double-blind, case-control, and prospective studies. Although these studies are heterogeneous in terms of design, intervention methods, and measures of outcome, bias can be reduced using statistical analysis strategies.

Some studies included in our review address tailored interventions [11, 26, 39]. According to Cohen-Mansfield [19], dementia patients become agitated when their needs are not perceived or addressed by caregivers. These needs can be addressed by a “person-centered care model.” Tailored interventions are currently being considered as more effective than standardized interventions. Garland et al. [26] reported that audiotapes containing a family member's voice were more effective than audiotapes with songs to reduce agitation in patients with BPSD. Gitlin et al. [48] and O'Connor et al. [11] described a home-based occupational therapy program based on personal capabilities and individual preferences. This tailored program promoted a significantly greater reduction in agitation. In a randomized controlled trial, Gitlin et al. [48] found that nonpharmacological interventions based on Tailored Activities Program are cost-effective and should be considered as part of the clinical management of dementia.

## 5. Conclusion

Studies focusing on alternative approaches have disclosed that different nonpharmacological interventions are able to provide positive results in reducing symptoms of BPSD. Most studies have demonstrated that these interventions have important and significant efficacy improving BPSD such as agitation, psychotic symptoms, and apathy. Undesired side effects of pharmacological treatments, as antipsychotics and benzodiazepines, have promoted a search for alternative treatments for BPSD. Therefore, nonpharmacological interventions programs should be considered as first-option interventions to treat BPSD.

## Figures and Tables

**Table 1:** Summary of nonpharmacological interventions studies to reduce behavioral and psychological symptoms of dementia (BPSD).

Author	Country	Intervention	*n*	Place	Assessment	Symptoms treated	Comments
(1) Woods and Dimond (2002) [5]	(Canada) USA	Touch therapy	57	Long term care facilities	(i) Agitated Behaviors Rating Scale-modified (ii) Memory and Behavior Checklist	Manual manipulation (restlessness) and vocalization	

(2) Holmes et al. (2006) [24]	UK	Music therapy	32	Home care or nursing home facility	DCM	Apathy	

(3) Svansdottir and Snaedal (2006) [25]	Iceland	Music therapy	38	Nursing homes and geriatric wards	BEHAVE-AD	Agitation, aggressiveness, and anxiety	Positive effects mostly disappeared 4 weeks after the end of intervention

(4) Garland et al. (2007) [26]	Australia	Simulated family presence/preferred music	30	Nursing home		Shadowing and repetitive questioning	

(5) Lin et al. (2007) [27]	China	Aromatherapy	140	Care and attention homes	(i) Cohen-Mansfield Inventory (ii) NPI	Physically agitated behaviors and verbally agitated behaviors	

(6) Gitlin et al. (2008) [28]	USA	Activities program	60	Community	(i) Agitated Behaviors in Dementia Scale (ii) Revised Memory and Behavior Problem Checklist	Shadowing and repetitive questioning Agitation Argumentative behaviors	

(7) Burns et al. (2009) [29]	UK	Light therapy	48	Nursing care setting	Cohen-Mansfield Inventory	Physically agitated behaviors Sleep disturbance	

(8) Cerga-Pashoja et al. (2010) [22]	UK	Physical exercises	146	Community-dwelling individuals	NPI	BPSD in general	

(9) van der Ploeg et al. (2010) [30]	Australia	Activities		Aged care facilities	Cohen-Mansfield Inventory	Physically agitated behaviors	

(10) Burns et al. (2011) [31]	UK	Aromatherapy	81	Care homes	(i) Pittsburgh Agitation Scale (ii) NPI	Agitation	No statistical difference between melissa aromatherapy × medication × placebo

(11) Kolanowski et al. (2011) [32]	USA	Activities program	128	Nursing homes	(i) Cohen-Mansfield Inventory (ii) Passivity in Dementia Scale	Agitation and anxiety	Findings indicate that activities customized may help reduce agitation and passivity throughout the day and not just during the treatment

(12) Sung et al. (2012) [33]	Taiwan	Music therapy	60	Home care facility	(i) Cohen-Mansfield Inventory (ii) Rating of Anxiety in Dementia	Anxiety and wellbeing	

(13) O'Connor et al. (2014) [11]	Australia	Activities program	160	Community	NPI-C	—	No results (protocol study)

(14) Brunelle-Hamann et al. (2015) [34]	Canada	Cognitive rehabilitation	15	—	NPI-12	Delusions	Small sample might have limited the power of the study and increased the likelihood of type 1 errors

(15) Chen et al. (2014) [23]	Taiwan	Combination of nonpharmacological interventions	92	Residential care facility	NPI	Hallucinations, delusion, and agitation	Not randomized controlled design The increased interaction between participants and the research staff might have caused a confounding effect in the “therapeutic” effects

(16) Lowery et al. (2014) [15]	UK	Physical exercises	131	Community mental health or primary clinical service	NPI	BPSD, except hallucinations and delusions	Physical exercise tailored to participant-carer dyads had no significant effect in BPSD but had statistical difference in caregiver burden

(17) Neville et al. (2014) [35]	Australia	Physical exercises (aquatic exercises)	11	Residential aged care facilities	(i) The Psychological Wellbeing in Cognitively Impaired Persons Scale (ii) Revised Memory and Behavior Problem Checklist	Psychological wellbeing	Small sample size No control group

(18) O'Connor et al. (2014) [11]	Australia	Activities	180	Community	NPI-C	—	No results (protocol study)

(19) Dowling et al. (2007) [36]	USA	Light therapy	70	Nursing homes	NPI	Agitation/aggression, depression/dysphoria, aberrant motor behavior, and appetite/eating disorders	

(20) Yang et al. (2015) [21]	Taiwan	Aromatherapy	189	Retirement homes for veterans and long term care facilities	Cohen-Mansfield Inventory	Agitation	

DCM: Dementia Care Mapping; NPI: Neuropsychiatric Inventory; NPI-C: Neuropsychiatric Inventory Clinician; BEHAVE-AD: Behavioral Pathology in Alzheimer's Disease Rating Scale.
